# Creep Resistance of S304H Austenitic Steel Processed by High-Pressure Sliding

**DOI:** 10.3390/ma15010331

**Published:** 2022-01-03

**Authors:** Petr Kral, Jiri Dvorak, Vaclav Sklenicka, Zenji Horita, Yoichi Takizawa, Yongpeng Tang, Lubomir Kral, Marie Kvapilova, Pavla Roupcová, Jakub Horvath

**Affiliations:** 1 Institute of Physics of Materials, Czech Academy of Sciences, Zizkova 22, 616 00 Brno, Czech Republic; dvorak@ipm.cz (J.D.); vsklen@ipm.cz (V.S.); lkral@ipm.cz (L.K.); kvapilova@ipm.cz (M.K.); roupcova@ipm.cz (P.R.); 2 Kyushu Institute of Technology, Kitakyushu 804-8550, Japan; horita.zenji.688@m.kyushu-u.ac.jp (Z.H.); tang.yongpeng316@mail.kyutech.jp (Y.T.); 3 Magnesium Research Center, Kumamoto University, Kumamoto 860-8555, Japan; 4 Synchrotron Light Application Center, Saga University, Saga 840-8502, Japan; 5 Technology Department, Nagano Forging Co., Ltd., Nagano 381-0003, Japan; ytakizawa@nsc-com.co.jp; 6 UJP PRAHA a.s., 156 10 Praha-Zbraslav, Czech Republic; horvathj@ujp.cz

**Keywords:** austenitic steels, creep properties, severe plastic deformation

## Abstract

Sheets of coarse-grained S304H austenitic steel were processed by high-pressure sliding (HPS) at room temperature and a ultrafine-grained microstructure with a mean grain size of about 0.14 µm was prepared. The microstructure changes and creep behavior of coarse-grained and HPS-processed steel were investigated at 500–700 °C under the application of different loads. It was found that the processing of S304H steel led to a significant improvement in creep strength at 500 °C. However, a further increase in creep temperature to 600 °C and 700 °C led to the deterioration of creep behavior of HPS-processed steel. The microstructure results suggest that the creep behavior of HPS-processed steel is associated with the thermal stability of the SPD-processed microstructure. The recrystallization, grain growth, the coarsening of precipitates led to a reduction in creep strength of the HPS-processed state. It was also observed that in the HPS-processed microstructure the fast formation of σ-phase occurs. The σ-phase was already formed during slight grain coarsening at 600 °C and its formation was enhanced after recrystallization at 700 °C.

## 1. Introduction

The S034H steel is austenitic 18 wt.% Cr–9 wt.% Ni stainless steel with the addition of about 3 wt.% Cu-forming nano-sized Cu precipitates [1,2]. This steel exhibits good creep strength, high ductility, and corrosion resistance [3,4,5,6]. For this reason, S304H steel is used for high-temperature components of advanced power plants such as boiler, reheater, and superheater tubes [2,7]. 

The strength of S304H steel in the recrystallized state is not high at room temperature [6,8]. However, it can be significantly improved by work hardening and/or grain refinement. Previous works investigating the effect of severe plastic deformation (SPD) on the mechanical properties revealed that the ultimate tensile strength (UTS) is improved approximately three times and yield strength (YS) about seven times [9] in comparison with annealed coarse-grained (CG) state. A significant increase in strength was observed also in other SPD-processed austenitic stainless steels such as 304L and 316L. It was found that short-term annealing up to 600 °C of SPD-processed S304H steel does not cause a significant decrease in strength at RT. However, the RT strength is significantly reduced after short-term annealing at 700 °C [10]. 

The large strains imposed into the microstructure can also lead to phase transformation [11,12,13]. It was found that initially austenitic microstructure in stainless steels can be partially or fully transformed into strain-induced martensite [9,10,14,15,16]. This effect was used in previous studies for the production of ultrafine-grained austenitic steels through reversion transformation from strain-induced martensite [17,18]. However, the previous studies showed that the large strains imposed into the austenite steels at RT do not always lead to the transformation of austenite into the strain-induced martensite. It was observed that cold rolling, high-pressure torsion, and multiple forging at RT led to the transformation of austenite into the strain-induced martensite [9,10,14,15,16]. However, there are also studies that have shown that SPD does not lead to the formation of the measurable amount of strain-induced martensite in austenitic steels [19,20]. 

Fine-grained microstructures can also be found in real components such as surface layers of tubes processed by shot peening [21]. The large plastic strains imposed by shot peening and subsequent heating lead to the microstructure refinement. In the fine-grained microstructure, there is the enhanced diffusion of chromium and the formation of chromium oxides on the tube surface layers. However, it was also found that plastic deformation of austenitic steels leads to the fast formation of σ-phase [22,23]. The σ-phase is intermetallic tetragonal phase with a = 0.879 nm and c = 0.456 nm. This phase is very brittle and its formation in the microstructure leads to a decrease in mechanical properties (such as ductility and impact energy) as well as corrosion resistance in CG austenitic steels due to the depletion of Cr from the microstructure [5,24]. The thickness of surface layers processed by shot peening is usually about 400 µm [21]. For this reason, the production of creep specimens from surface layers and thus the investigation of their creep behavior is difficult. Presently, there are only limited numbers of studies investigating creep behavior and microstructure changes in austenitic steels after application of large plastic strains [16]. The SPD methods provide a sufficient amount of experimental material to study the influence of large plastic strains on creep behavior and microstructure changes in S304H, which may be similar to those found in the surface layers of tubes. Thus, SPD-processed austenitic steels can be considered not only as materials for basic research, but the results in the present study may contribute to the clarification of creep and microstructural processes occurring in certain locations of high-temperature components such as surface layers of tubes. The aim of this study is to investigate the influence of creep testing temperature on creep resistance and microstructure of HPS-processed S304H steel.

## 2. Materials and Methods

A standard chromium-nickel stainless steel S304H was used as a coarse-grained (CG) state. The CG state was received in the form of a thin-walled pipe with an outer diameter of about 38 mm and a thickness of about 6.8 mm. During continuous production of the pipe, the pipe was subjected to solution annealing at 1150 °C/2 min. and cooled with a water shower. The chemical composition is shown in Table 1. The chemical composition of CG state was determined by energy dispersive spectroscopy (EDS) in Tescan Lyra 3 XMU scanning electron microscope (SEM) as the mean value of 10 measurements.

The sheets with dimensions 100 × 10 × 1 mm were manufactured parallel to the pipe axis. The sheets of CG state were subsequently processed by high-pressure sliding (HPS) technique at room temperature under a pressure of 4 GPa with a sliding distance of 15 mm. The equivalent strain imposed into the material was about 7.8 [25,26].

The creep specimens of CG and HPS-processed S304H steel with a gauge length of 8 mm and cross-section of 3 × 1 mm^2^ were used for tensile creep testing under constant load. Tensile creep tests were performed at different stresses and temperatures of 500 °C, 600 °C, and 700 °C. 

The microstructure was studied using Tescan Lyra 3 XMU (Tescan, s.r.o., Brno, Czech Republic) scanning electron microscope (SEM) equipped by electron backscatter diffraction (EBSD) and transmission electron microscope (TEM) Jeol 2100F (JEOL Ltd, Tokyo, Japan) operated at 200 kV. The surface of specimens for EBSD investigation was ground with SiC papers up to 4000, subsequently polished with a colloidal silica suspension (OP-S), and then electrolytically polished using an electrolyte consisting of 10% perchloric acid and 90% acetic acid. The high-angle grain boundaries (HAGBs) were characterized as boundaries with misorientation angle θ ≥ 15° and low-angle grain boundaries (LAGBs) as boundaries with θ < 15°. 

The specimens for TEM were prepared by Tenupol 5 (Struers Inc., Cleveland, OH, USA) twin-jet electrolytic polishing unit using an electrolyte consisting of 5% perchloric acid (HClO_4_) and 95% acetic acid (CH_3_CO_2_H). Phase identification in TEM was performed by energy dispersive spectroscopy (EDS) and selective area electron diffraction (SAED). EBSD investigations were conducted at accelerating voltage of 20 kV with a specimen tilt of 70° from horizontal on areas of minimal dimensions of 20 × 16 µm with a step size 0.05 µm. The HPS-processed specimen was investigated by t-EBSD [27] with step size of 0.01 µm at 30 kV using TEM foil tilted at 20° from horizontal. The microstructure was also investigated by X-ray diffraction (XRD) at room temperature (RT) using diffractometer Empyrean (Malvern Panalytical) with CoKα radiation.

## 3. Results

### 3.1. Effect of HPS on Microstructure of S304H

**Figure 1:**
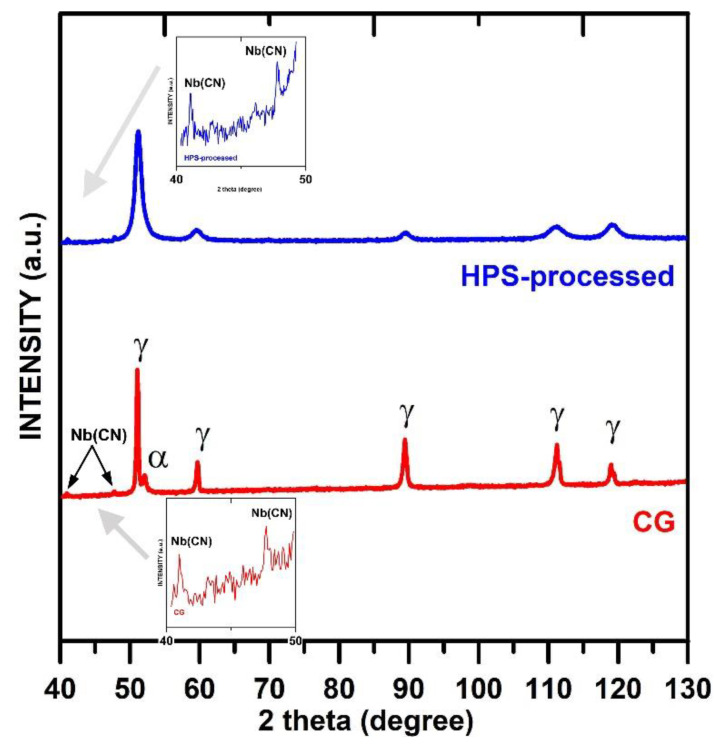
X-ray diffractograms obtained for coarse-grained and HPS-processed S304H steel.

Figure 1 shows XRD diffraction patterns for CG and HPS-processed S304H steel. The results demonstrate that microstructure both CG and HPS-processed state contains predominantly FCC austenite phase with a small portion of NbC precipitates (up to 1%).

The XRD result shows small peaks for the BCC phase in the CG state indicating that there may be about 4.5% of the BCC phase in the CG state. However, no peaks for strain-induced martensite are seen in the HPS-processed state. This result suggests that the content of possible BCC strain-induced martensite is low in the HPS state to be detected by this method. 

**Figure 2:**
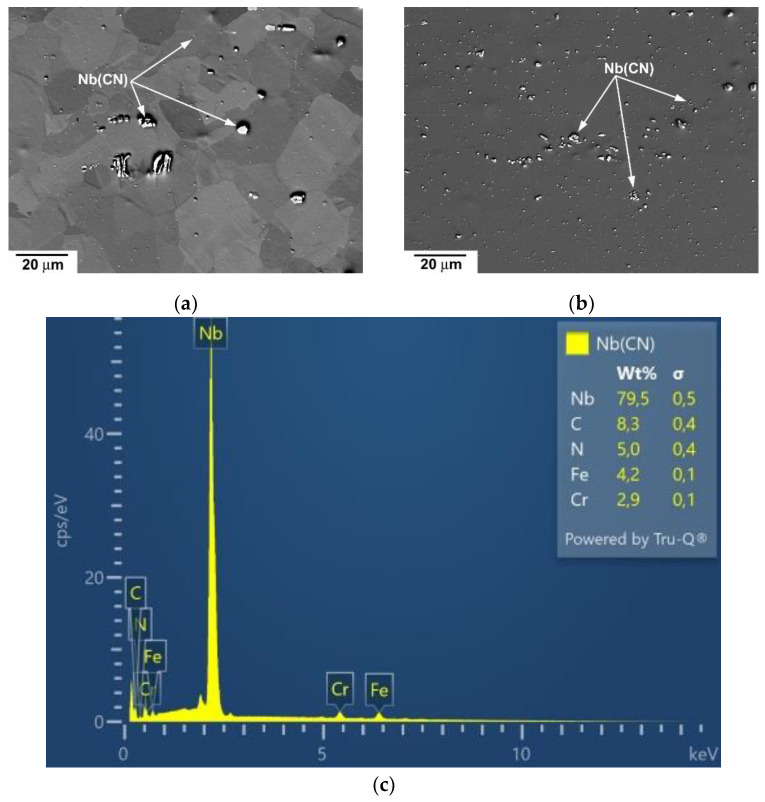
Nb carbonitrides in microstructure of S304H steel before creep testing (**a**) CG state, (**b**) HPs-processed state and (**c**) chemical composition of Nb carbinitrides.

Figure 2 demonstrates the distribution and selected chemical composition of Nb(CN). One can see that in the microstructure of CG state (Figure 2a) can be found large Nb(CN) exceeding the size of 3 µm and fine Nb(CN) with the mean size of about 0.1 µm. The mixture of large and fine Nb(CN) is also seen in the microstructure of HPS-processed S304H steel. The results demonstrate that HPS processing led to the fragmentation of large particles into smaller ones.

**Figure 3:**
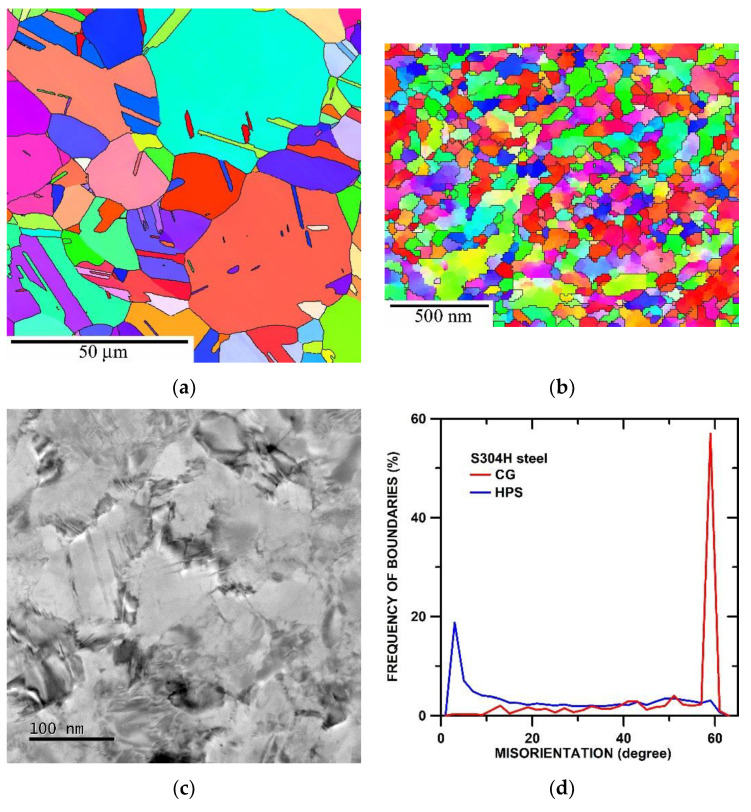
Microstructure of S304H steel before creep testing (**a**) inverse pole figure (IPF) for CG state, (**b**) IPF for HPS-processed state, (**c**) TEM micrograph of HPS-processed state and (**d**) misorientation distributions for CG and HPS-processed state.

Figure 3a shows that the grains with a mean size of about 8 µm and subgrains with a mean size of about 5 µm. In the microstructure was found about 95% of HAGBs and a large portion of boundaries was identified as Σ3 (111/60°) boundaries (Figure 3d). The application of HPS led to a significant reduction in mean grain size down to about 0.14 µm (Figure 3b). In the HPS-processed state was measured about 58% of HAGBs (Figure 3d). TEM micrographs (Figure 3c) showed that the boundaries are blurred due to high internal stresses (distortion of lattice near the boundaries). In the microstructure, the very fine deformation twins in the interior of the deformed grains can sporadically be found. 

### 3.2. Ductility and Creep Resistance of CG and HPS-Processed S304H States

**Figure 4:**
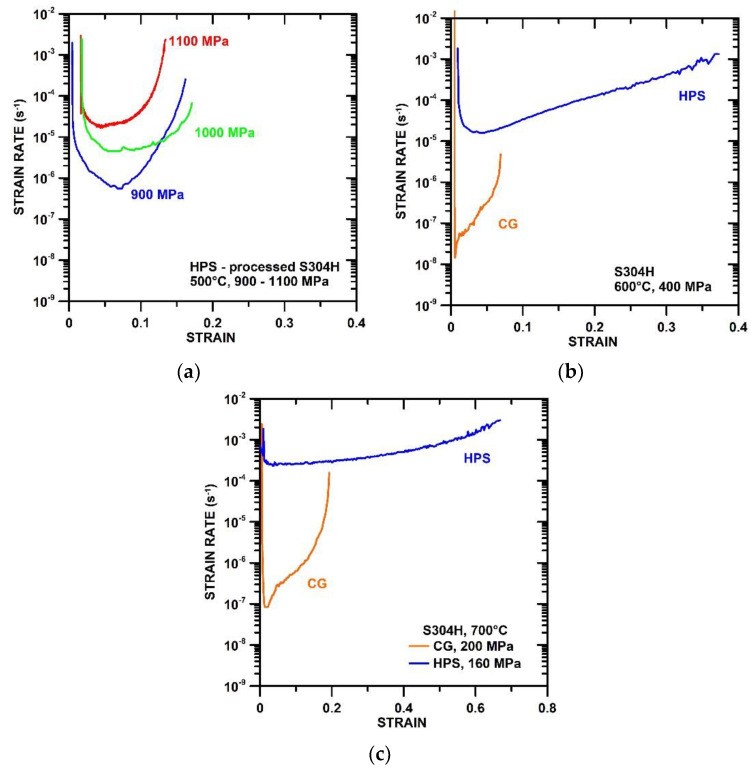
Strain rate vs. strain dependences measured at (**a**) 500 °C for HPS-processed state, (**b**) 600 °C for CG and HPS-processed states and (**c**) 700 °C for CG and HPS-processed state.

Figure 4a–c shows the dependences of the strain rate against strain. The creep results show that strain to fracture is significantly higher in the HPS-processed state in comparison with the CG state. It is seen that strain to fracture decreases significantly with decreasing testing temperature when the creep tests with similar minimum creep rates (${\dot{\varepsilon }}_{min}$) are compared. The creep results also demonstrate that the length of the primary stage in the HPS-processed state is relatively long in the specimen tested at 500 °C. However, the length of the primary stage decreases with increasing testing temperature.

The CG S304H steel exhibits short primary stadium and ${\dot{\varepsilon }}_{min}$ occurred up to strain about 0.02. The largest part of creep strain in HPS and also CG state tested at 600 °C and 700 °C occurs during the tertiary stage. 

### 3.3. Stress Dependences of Minimum Creep Rates for CG and HPS-Processed States

**Figure 5:**
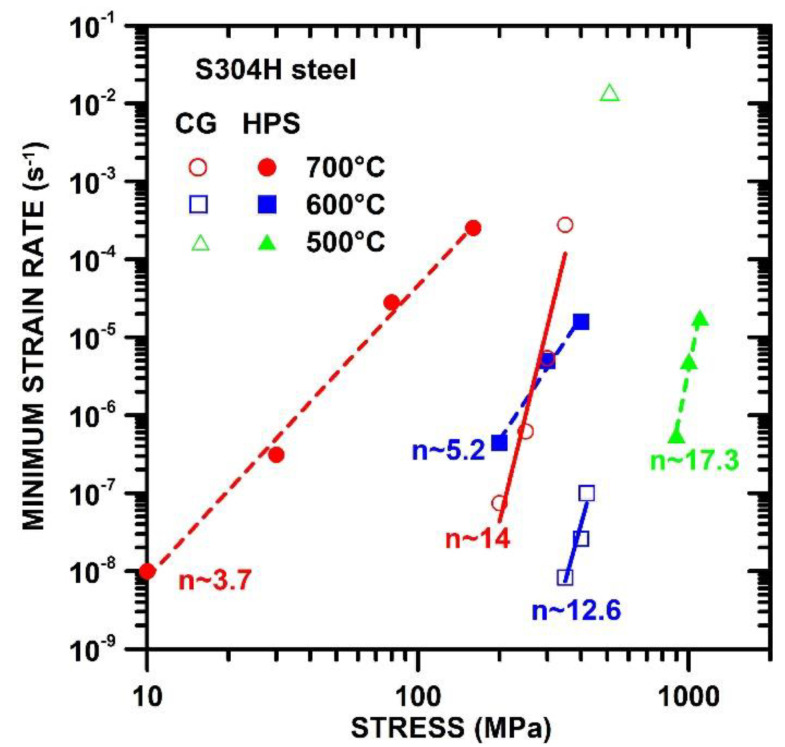
Stress dependences of minimum creep rates determined at 500 °C, 600 °C and 700 °C for CG and HPS-processed state.

Figure 5 shows the dependences of minimum creep rates against applied stress determined at 500 °C, 600 °C, and 700 °C. The results demonstrate that the ${\dot{\varepsilon }}_{min}$ measured at 600 °C and 700 °C for the HPS-processed state are significantly faster in comparison with the CG state. One can see that the differences in minimum creep rates measured for the HPS-processed and CG states decrease with the increasing value of applied stress. Thus, the ${\dot{\varepsilon }}_{min}$ measured for the CG state approaches the ${\dot{\varepsilon }}_{min}$ measured for HPS-processed state as the applied stress increases. The values of stress exponents of minimum creep rate $n=dln{\dot{\varepsilon }}_{min}/dln\sigma$ for HPS-processed state are about 5.2 determined at 600 °C and 3.7 determined at 700 °C. The values of stress exponents *n* measured for the HPS-processed state at 600 °C and 700 °C are significantly lower than that determined for the CG state at similar creep testing conditions. 

The creep tests performed at 500 °C showed that the creep behavior of the HPS and CG states is too different that it was not possible to perform the creep tests at the same stress interval. The creep results measured at 500 °C demonstrate that the minimum creep rates of the HPS-processed state determined at stresses between 900–1100 MPa were significantly slower than the minimum creep rate for CG state measured at 510 MPa. This is the opposite result to that found at 600 °C and 700 °C. The results for the HPS-processed state measured at 500 °C also demonstrate that the decrease in testing temperature led to a significant increase in stress exponent *n* to the value about 17.

### 3.4. Microstructure of HPS-Processed S304H Steel after Creep Testing

**Figure 6:**
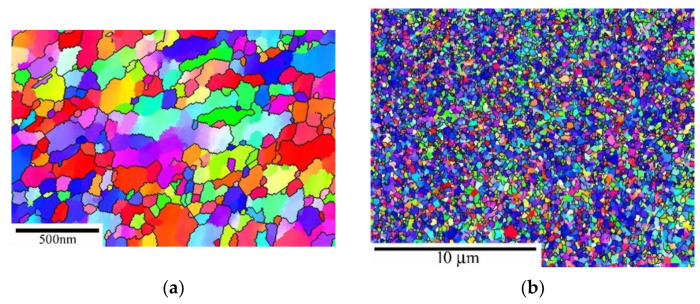

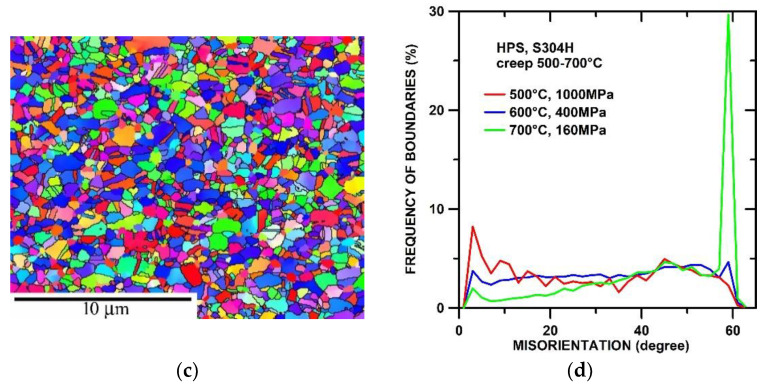
Microstructure of HPS-processed S304H steel observed in gauge length after creep testing at (**a**) 500 °C and 1000 MPa, (**b**) 600 °C and 400 MPa, (**c**) 700 °C and 160 MPa and (**d**) misorientation distributions measured at different testing temperatures after short-term creep.

Figure 6 shows the changes in the HPS-processed microstructure with increasing testing temperature after short-term creep testing. The t-EBSD results (Figure 6a) demonstrate that the short-term creep testing at 500 °C (t_f_~5.3 h) did not lead to significant grain coarsening and changes in misorientation distribution compared to the HPS-processed state before creep testing (Figure 3b,d).

The mean grain size is about 0.15 µm and in the microstructure was found about 71% HAGBs. However, during creep testing at 600 °C and 400 MPa (t_f_~1.8 h), the coarsening of grains was observed (Figure 6b). The mean grain size in the gauge length of the tensile specimen was about 0.29 µm. The misorientation distribution demonstrates that creep testing at 600 °C led to the decrease in LAGBs and in the microstructure was about 82% of HAGBs. The largest changes in the HPS-processed microstructure occurred during creep testing at 700 °C (Figure 6c,d). One can see that the short-term creep testing at 700 °C and 160 MPa (t_f_~0.42 h) led to the recrystallization of the microstructure (Figure 6c). The mean grain size in gauge length of this specimen was about 0.45 µm and in the microstructure was measured about 93% of HAGBs (Figure 6d). The microstructure contains a high number (about 35%) of Σ3 twin boundaries (111/60°).

### 3.5. Formation of Precipitates in HPS-Processed S304H Steel during Creep Testing

**Figure 7:**
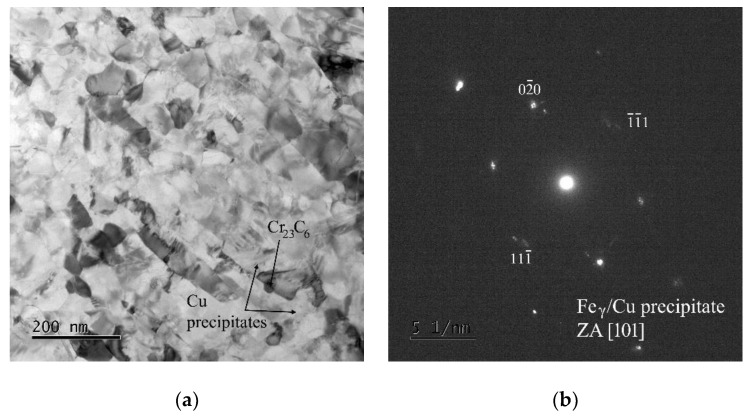

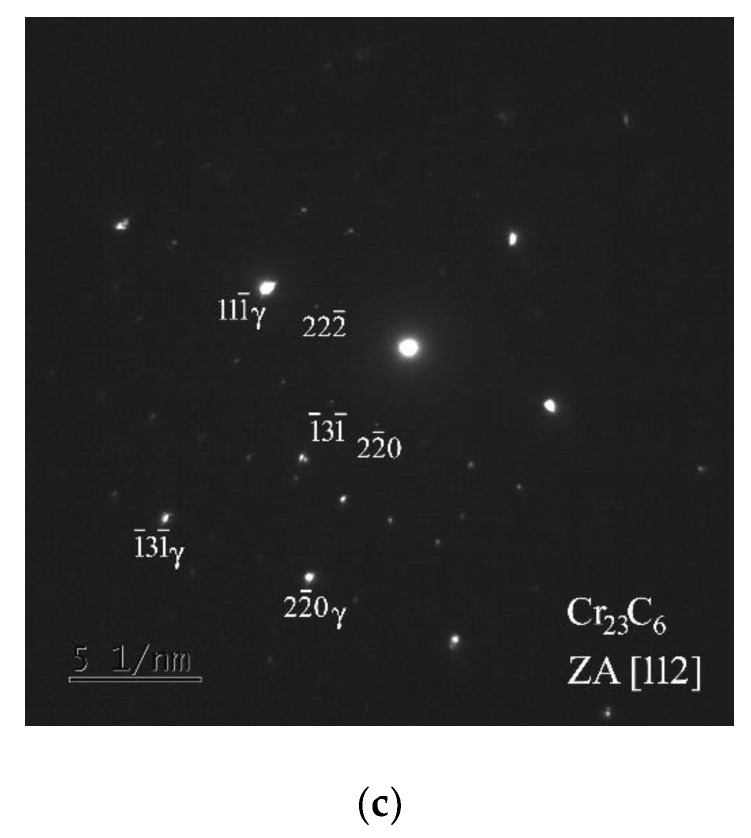
(**a**) TEM micrograph for HPS-processed steel tested at 500 °C and 1000 MPa, (**b**) diffraction pattern of austenitic matrix and fine coherent Cu precipitates, (**c**) diffraction pattern of Cr_23_C_6_ carbide.

Figure 7 shows the microstructure of the specimen tested at 500 °C and 1000 MPa. The TEM micrograph and diffraction patterns demonstrate that in the microstructure after short-term creep testing at 500 °C can be found very fine coherent Cu precipitates and Cr_23_C_6_ carbides.

**Figure 8:**
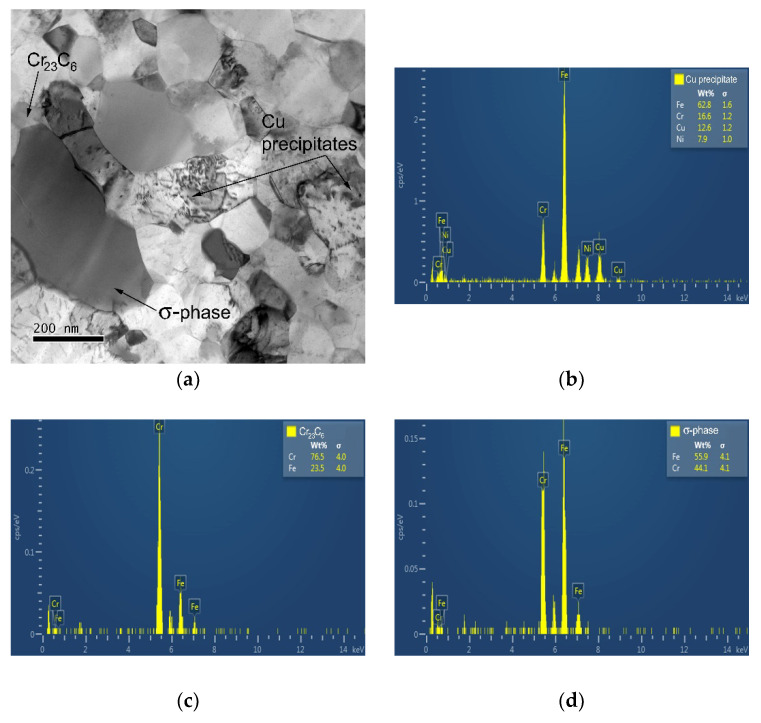
TEM micrographs for HPS-processed steel tested at (**a**) 600 °C and 400 MPa, (**b**) chemical composition of Cu precipitate, (**c**) chemical composition of Cr_23_C_6_ carbide and (**d**) chemical composition of σ-phase.

The HPS-processed microstructure after creep testing at 600 °C and 400 MPa is shown in Figure 8a. One can see that creep testing at 600 °C led not only to the grain coarsening but also to the formation of σ-phase along the grain boundary and at Cr_23_C_6_ carbide interface and to the coarsening of Cu precipitates. Figure 8b–d shows the chemical composition (point analysis) of the Cu precipitate, Cr_23_C_6_ carbide, and σ-phase. The chemical composition of the fine Cu precipitate is significantly influenced by the chemical composition of the matrix.

**Figure 9:**
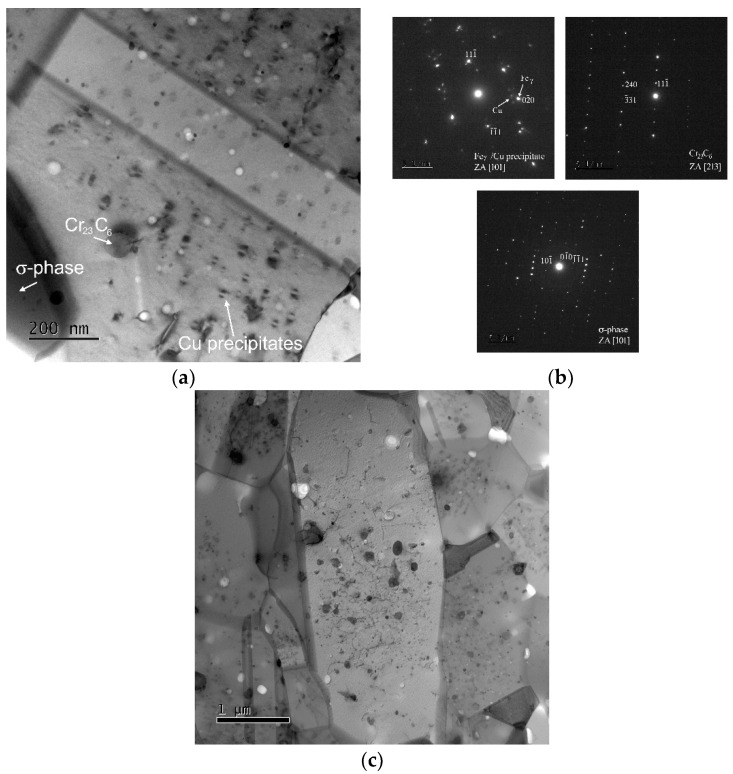
TEM micrographs for HPS-processed steel tested at (**a**) 700 °C and 30 MPa, ε = 0.1, creep time ~ 65 h; (**b**) diffraction patterns of Cu precipitate, Cr_23_C_6_ carbide and σ-phase, (**c**) 700 °C and 30 MPa, ε = 0.4, creep time ~ 235 h.

The microstructure of HPS-processed S304H steel tested at 700 °C and 30 MPa is shown in Figure 9a,b. The results demonstrate that there are fine Cu precipitates in the interiors of grains. The Cu precipitates are observed with coffee-bean contrast with a null contrast line indicating the coherent nature of the precipitates [28,29]. In the microstructure were also observed Cr_23_C_6_ carbides and σ-phase. The comparison of microstructures observed in the specimens tested at 700 °C under 400 MPa and interrupted in creep strain at about 0.1 and 0.4 revealed that the grains coarsen and the number of precipitates in the grain interiors decreases with increasing creep strain and time. It is also seen that the dissolution of the precipitates is more pronounced along the grain boundaries. In the specimen tested at 700 °C/30 MPa and interrupted at creep strain about 0.4, the free precipitates zones are seen near the boundaries.

**Figure 10:**
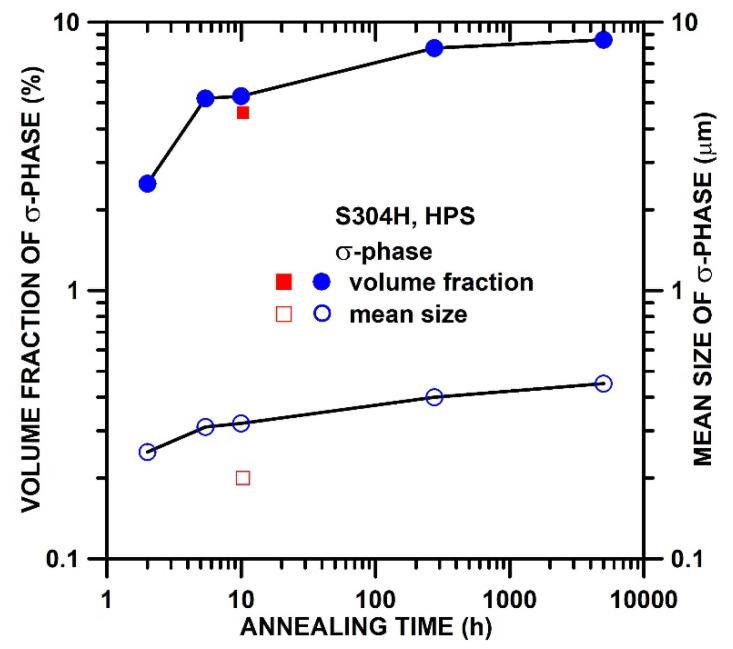
Mean size and amount of σ-phase in HPS-processed microstructure after static annealing at 700 °C and 600 °C. Blue symbols are for 700 °C and red symbols for 600 °C.

Figure 10 shows the amount and mean size of the σ-phase in the HPS-processed state after static annealing at 700 °C and in the stress-free grip parts of creep specimens determined by EBSD. It should be noted that it is necessary to add 5 h for heating to the test temperature to the time to fracture. The microstructure results demonstrate that the grip part of the HPS-processed specimen tested at 600 °C and 400 MPa with annealing time of about 6.8 h contains about 4.6% of σ-phase with a mean size of about 0.2 µm.

However, more σ-phase was formed during static annealing at 700 °C. During annealing for 2 h is formed about 2.5% of σ-phase with a mean size of about 0.25 µm. The amount and size of the σ-phase increase significantly during the first 10–100 h and then the growth rate with further increasing time of static annealing slows down significantly.

## 4. Discussion

It is generally accepted that severe plastic deformation leads to grain refinement. However, during SPD other effects such as phase transformations or the formation/dissolution of precipitates may occur. It should be noted that the precipitates can be fragmented during the SPD process. The occurrence of precipitate fragmentation during SPD was reported in Al-Cu, Al-Mg-Si, and Al-7050 [30,31,32]. The refinement of Nb(CN) through the fragmentation of the precipitates during HPS was also observed in this work. A common phenomenon in SPD of austenitic steels is the transformation of the austenite phase into the strain-induced martensite. However, this phase transformation does not always occur [19,20]. In the present work, the formation of strain-induced martensite was also not observed. The absence of a measurable amount of strain-induced martensite can be caused by the slight heating of the specimen during HPS. Previously published works [14,20] showed that the use of the same processing method and the same material can lead to different results with respect to strain-induced martensite formation. This means that not only the SPD technique used for specimen processing but also other details of the severe deformation such as the speed of plastic deformation, size of the specimen, and related slight heating of the specimen during SPD can significantly influence phase-transformation during SPD. The present results showed that the short-term creep testing at 500 °C led to the formation of very fine Cu precipitates in the HPS-processed state. These precipitates can significantly contribute to a high creep strength of the HPS-processed state at 500 °C. The significant increase in the strength of alloys after the application of low-temperature annealing after SPD treatment in comparison with their CG counterparts was also observed in previous works [33,34].

Previous works [35,36] investigating the formation and fast growth of σ-phase is associated with recrystallization. In the present work, it was observed that the σ-phase is formed also during slight coarsening of grains before recrystallization occurs. Recently, it was suggested [23] that the formation of the σ-phase can occur before the recrystallization due to the release of the high local stress concentrations at triple junctions or phase boundaries of Nb carbides. This recently found mechanism can explain the precipitation of σ-phase at 600 °C in the non-recrystallized microstructure of HPS-processed S304H steel.

The σ-phase formation at elevated temperature before recrystallization may therefore be related to the recovery of crystallographic defects density causing high internal stresses and to the reduction in the number of non-equilibrium boundaries forming long-range elastic stresses [14,37,38,39].

The high density of free dislocations in the grain interior leads to the extended strain during the primary stage [40]. The creep results for the HPS-processed state showed that the higher the creep testing temperature the shorter the primary stage (Figure 4). This result suggests that the density of free dislocations in the HPS-processed state decreases with increasing testing temperature.

Based on the length of the primary stage strain, it can be suggested that after the recrystallization at 700 °C in the HPS state is the density of free dislocations is similarly low as the density of free dislocations in the annealed CG state. However, the ${\dot{\varepsilon }}_{min}$ of the HPS-processed state is significantly faster compared to the CG state. The low creep strength and high ductility of the HPS-processed state are associated with a high number of HAGBs which play an important role in the softening and improvement in ductility at 600 °C and 700 °C. Similar creep behavior was also observed in the other SPD-processed alloys [41] and was explained by grain-boundary mediated processes such as grain-boundary sliding, enhanced recovery of dislocations, or diffusion creep [42,43,44]. The creep mechanism associated with grain boundaries is significantly influenced by grain boundary diffusion, which can be markedly faster than in CG materials [45,46]. The rapid σ-phase formation at 600 °C and 700 °C associated with the diffusion of Cr and the observation of precipitation free-zones facilitating the dislocation movement near boundaries show that diffusion in HPS state is very fast.

However, opposite creep results were found when HPS-processed steel was tested at 500 °C. The HPS-processed state exhibited significantly higher creep strength than CG steel. Similar tensile strength for S304H steel at 500 °C was also found in other work [6]. It can be suggested that the high creep strength of HPS-processed S304H steel at 500 °C is caused by the partial preservation of the SPD microstructure formed at RT up to the temperature of 500 °C and the formation of very fine Cu precipitates. Thus, the combination of very fine grain size and precipitates, high number of LAGBs, high dislocation density in grain interiors, and the presence of non-equilibrium boundaries provide high creep resistance of HPS-processed state at 500 °C and high stresses. However, in the case that SPD-processed material is shorty annealed before creep testing and the density of crystallographic defects inside of the grains is reduced, the decrease in the strength of SPD material occurs [10,47].

## 5. Conclusions

The austenitic S304H steel was processed by high pressure sliding at room temperature and the effect of SPD on creep behavior and microstructure changes at 500–700 °C was investigated.

The following conclusions can be made:

1. HPS-processed S304H steel exhibits markedly high creep strength at 500 °C. However, the creep strength of the HPS-processed state decreases at creep temperatures of 600 °C and 700 °C compared to the CG state.
2. The creep strength of HPS-processed steel is associated with the coarsening of SPD-processed microstructure and the formation of very fine precipitates. The coarsening of precipitates, recrystallization, and grain growth leads to the reduction in creep strength of the HPS-processed state.
3. In the HPS-processed S304H steel, the fast formation of σ-phase was observed. The σ-phase was formed during slight grain coarsening at 600 °C and its formation was enhanced after recrystallization at 700 °C.

## Figures and Tables

**Table 1:** Chemical composition of as-received CG state.

Element	Cr	Ni	Cu	Nb	N
wt.%	18 ± 0.38	15.5 ± 0.42	3 ± 0.26	0.4 ± 0.05	0.2 ± 0.03

## Data Availability

Data sharing is not applicable to this article.
